# Energy Retention in Thin Graphite Targets after Energetic Ion Impact

**DOI:** 10.3390/ma14216289

**Published:** 2021-10-22

**Authors:** Damjan Iveković, Petar Žugec, Marko Karlušić

**Affiliations:** 1 Ruđer Bošković Institute, Bijenička cesta 54, 10000 Zagreb, Croatia; damjan.ivekovic@irb.hr; 2 Department of Physics, Faculty of Science, University of Zagreb, Bijenička cesta 32, 10000 Zagreb, Croatia

**Keywords:** graphite, ion irradiation, swift heavy ion, radiation hardness, ion track, Geant4

## Abstract

High energy ion irradiation is an important tool for nanoscale modification of materials. In the case of thin targets and 2D materials, which these energetic ions can pierce through, nanoscale modifications such as production of nanopores can open up pathways for new applications. However, materials modifications can be hindered because of subsequent energy release via electron emission. In this work, we follow energy dissipation after the impact of an energetic ion in thin graphite target using Geant4 code. Presented results show that significant amount of energy can be released from the target. Especially for thin targets and highest ion energies, almost 40% of deposited energy has been released. Therefore, retention of deposited energy can be significantly altered and this can profoundly affect ion track formation in thin targets. This finding could also have broader implications for radiation hardness of other nanomaterials such as nanowires and nanoparticles.

## 1. Introduction

Geant4 is a Monte Carlo toolkit for simulating passage of particles through matter. It is very versatile, used in many different areas of physics, such as high energy and nuclear physics [1]. By extending its scope to low energy physics, it has also found many uses in diverse applications such as hadron therapy, radiation processing of materials and devices, radiation shielding and evaluation of space radiation hazards [2,3,4]. For all these applications, and hadron therapy in particular, accurate calculation of ion ranges and ion energy losses is critically important [5]. 

While the total kinetic energy of the ion determines its range within the material, it is local energy deposited by the passing ion that causes material modification. This locally deposited energy is determined by the energy loss per unit path length of the energetic ion (*dE*/*dx*), which is usually modelled by the Bethe Bloch model with appropriate corrections at low and high energies. There are several codes such as SRIM [6], CasP [7] and PASS [8] that can calculate the ion energy losses in condensed matter exceptionally well [9]. This is important because impact of the energetic ion upon the material can produce damage only if ion energy loss exceeds a certain material-dependent threshold value. The main channel of the ion energy loss at these high energies (around 1 MeV per nucleon, i.e., 1 MeV/n) is the electronic energy loss by atomic ionisations, leading to dense electronic excitations along the ion path. Primary electrons generated this way, and subsequent cascades of secondary electrons, determine nanoscale cylinder-shaped volume of the material where material modifications can take place. At high energies, direct nuclear collisions are very rare and therefore nuclear energy loss is negligible. Although there are different models (thermal spike, Coulomb explosion, etc.) that aim to explain material modification processes following the ion impact, it is the electronic excitation in the first stage of the material modification process that has the most profound impact on the ion track formation, i.e., on the final, permanent damage in the material formed on the place of the ion impact [10]. 

Although the ion energy loss and deposited energy density are equal deep within the material, deposited energy density can be less than the ion energy loss close to the surface of the material. The reason for this can be found in the emission of the electrons from the surface [11] that can take away significant portion of the deposited energy [12]. One would expect this effect to make surface more resistant to material damage, but there are other competing processes occurring in later stages of ion track formation. For example, prompt recrystallisation can significantly reduce ion track size within the material [13] and cause threshold for ion track formation to be much higher than expected [14]. Since recrystallisation process is less efficient on material surface than in the bulk, it is the surface that could be more prone to ion induced damage. 

Effects of the high energy ion irradiation on very thin targets and 2D materials such as graphene are in the focus of research [15,16,17,18]. Material removal from later stages of the ion track formation in thin targets can lead to diverse ion track morphologies [18,19,20]. However, even more importantly, escape of the deposited energy into the vacuum via electron emission should substantially affect ion track formation in thin targets and 2D materials [18,21]. For example, in the case of 1 MeV/n silicon and xenon ions, graphene should retain only 50% and 60% of deposited energy, respectively [21].

The aim of the present work is to evaluate how much of deposited energy remains within the thin graphite targets. The present approach offers simple and practical solution to this problem. Using the Geant4 code, we have explored a wide range of ion types (from carbon up to xenon) and ion energies (0.1–10 MeV/n) interacting with graphite targets of different thicknesses (1–100 nm). More accurate results could be obtained with custom made [18] and much more computationally intensive codes [21]. In the case of graphene targets, one should also consider the atomic position where the ion impact occurs [21]. Results presented here offer broader view on this problem at the expense of avoiding extreme cases such as interactions of energetic ions with monolayer graphene. Thus, the range of irradiation parameters investigated in this work is very wide and covers most of typical ions and energies used in ion track experiments [22,23]. Graphite thicknesses also cover wide range of targets, from bulk graphite down to three-layer graphene. The present approach also opens up the opportunity to study the energy retention in other types of nanomaterials, such as nanoparticles and nanowires, as well as the energy transport across surfaces and interfaces.

## 2. Simulation Details

In our Geant4.10.05 simulations we defined a world (vacuum) 20 × 20 × 20 µm^3^ in size, containing graphite target of cylindrical shape, with diameter kept fixed at 1000 nm and thickness varied between 1–100 nm. The ion was created 10 nm away from the target, with trajectory perpendicular to its surface. Every simulation run consisted of 10^5^ ions being shot at the target. Different types of ions (C, O, Ne, Si, Ar, Fe, Kr, Xe) of varying kinetic energies (between 0.1–10 MeV/n) have been used (Table 1). In all cases, change in the ion kinetic energy after passage through the target was small. Consequently, a change in the ion electronic energy loss was negligible. For this reason, all ions passed through the target, and no ions were implanted within it. At these energies nuclear energy loss is very small. Values of simulation parameters are given in Table 1, together with values obtained with SRIM 2008 [6] and CasP 5.2 codes [7]. Very good agreement for the ion energy loss has been found. For the calculation of the ion energy losses, Geant4 uses ICRU73 values [24], available for all ions between lithium and argon (scaling for heavier ions is based on the effective charge approach) at energies between 0.025–10 MeV/n. Light ions such as protons, helium and lithium are excluded from this work because their electronic energy losses are very small and therefore insufficient for materials modifications. Moreover, we have found that for thin targets, many of these light ions pass through basically unaffected. 

By default, Geant4 models for the ion energy loss and delta-electron production by heavy charged particles use the effective charge approach, dynamically recalculating the ion effective charge in the material, based on its current speed. The rationale behind introduction of the effective charge is the electron exchange between the transported ion and the surrounding medium. In calculating it, a state of equilibrium between the ion and the medium is assumed. Geant4 offers the possibility of using a particular charge state, to be specified by user. To this end, the default models using the effective charge approach—G4BraggIonModel and G4BetheBlochModel—have to be replaced by G4BraggIonGasModel and G4BetheBlochIonGasModel variants. However, these models do not evolve the charge state further; rather, it is kept fixed at a specified value.

Only rarely have we found the production of secondaries other than electrons (for example photons). We have ignored them in our analysis because this channel of energy dissipation could be safely neglected. Electrons ejected from the target have also been monitored. Their number, energy and angle of emission have been evaluated. A threshold for a direct energy deposition by both the ion and the electrons has been set to 1 eV.

In this work, we use term *deposited energy* (*E_dep_*) for the energy deposited by the passing ion (and equal to the ion energy loss), and *retained energy* (*E_ret_*) for the energy that remained within the target material by the end of the simulation. The difference is equal to the *emitted energy* (*E_em_*) that was carried away into vacuum by the emitted electrons.

## 3. Simulation Results

**Figure 1:**
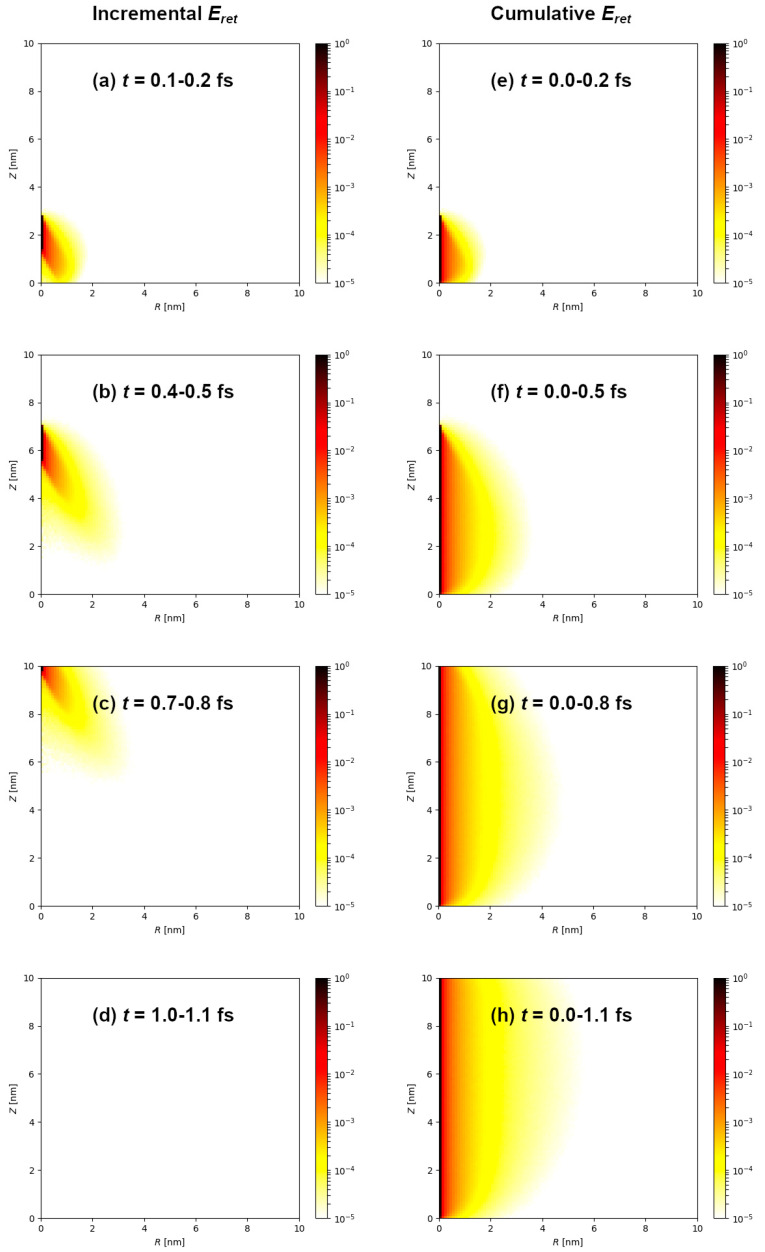
(**a**–**d**) Distribution of the retained energy density in cylindrical coordinates after 1 MeV/n Si ion impact into 10 nm thick graphite target. Incremental distributions are shown for several different times, as indicated on the panels, accumulated within 0.1 fs time window. (**e**–**h**) Cumulative retained energy density after 1 MeV/n Si ion passage through 10 nm thick graphite target, for different times as indicated on the panels.

Following the ion impact, electronic excitation spreads on the femtosecond timescale through the target material. The illustration shown in Figure 1a–d shows evolution of the electronic excitation during the passage of 1 MeV/n Si ion through 10 nm thick graphite target. The presented histograms show incremental distribution of the local retained energy density acquired within 0.1 fs, expressed in cylindrical coordinates (*R*, *z*) for the cylindrically shaped target:

(1) $$\frac{dE}{dV}=\frac{d^{3}E}{RdRd\varphi dz}$$

At this kinetic energy, velocity of the silicon ion is 1.4 × 10^7^ m/s and it traverses 10 nm thick target within 0.7 fs. This is clearly seen in Figure 1 as an electronic excitation front that originates and moves in the wake of the silicon ion. Even after the ion passage, electronic excitation is still evolving within the target. Furthermore, cumulative retained energy density is shown in Figure 1e–h. These graphs indicate the final spatial retained energy density distribution is reached already within 1 fs after the ion passage, mainly due to the slowing down of the primary (δ-ray) electrons, in agreement with previous works (Ref. [25] and references therein). 

**Figure 2:**
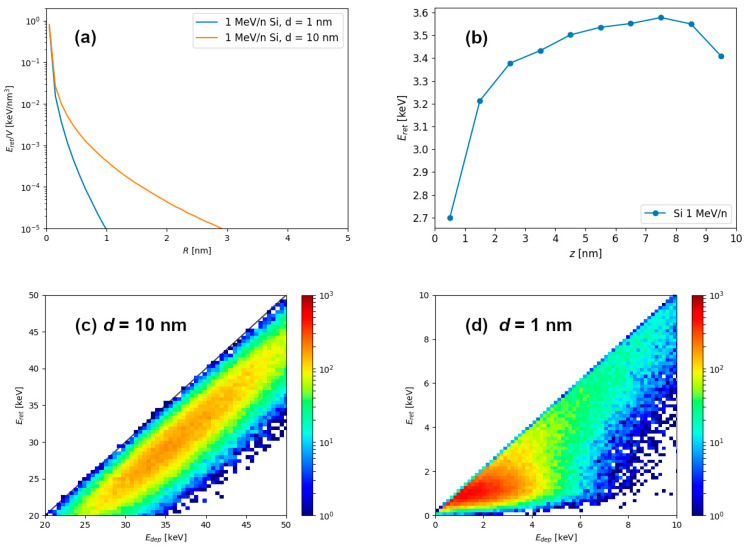
(**a**) Radial retained energy density profiles obtained after 1 MeV/n Si ion impact into the 1 nm thin target and the 10 nm thick target. (**b**) Depth profile of the retained energy within 10 nm thick target. Retained vs. deposited energy histograms, obtained after 10^5^ simulations using 1 MeV/n silicon ions and (**c**) 10 nm thick and (**d**) 1 nm thin target.

Simulation results of energetic ion passage through graphite (shown in Figure 1) can be used to calculate radial profiles of retained energy density shown in Figure 2a, as well as the depth profiles of retained energy shown in Figure 2b. Clearly, for the 1 nm thin target, larger percentage of the deposited energy is carried away outside the material via the electron emission, and consequently, less energy remains in the material. For this reason, retained energy density quickly diminishes at larger distances from the ion trajectory in the 1 nm thin target. In the case of the thicker target, such as the one shown here which has a thickness of 10 nm, retained energy density falls off slower at larger distances because the electrons from deeper within the material have less chance to escape from the target. 

Histograms of retained and deposited energies are shown for 1 MeV/n Si passage through 10 nm (Figure 2c) and 1 nm (Figure 2d) graphite targets. From this output we calculated that 84% of deposited energy remains within the material for the 10 nm thick target, and only 67% of deposited energy remains within 1 nm thin target. In both cases the dispersion of data was noticeable, but the retention of energy was clearly more pronounced for the 10 nm thick target.

**Figure 3:**
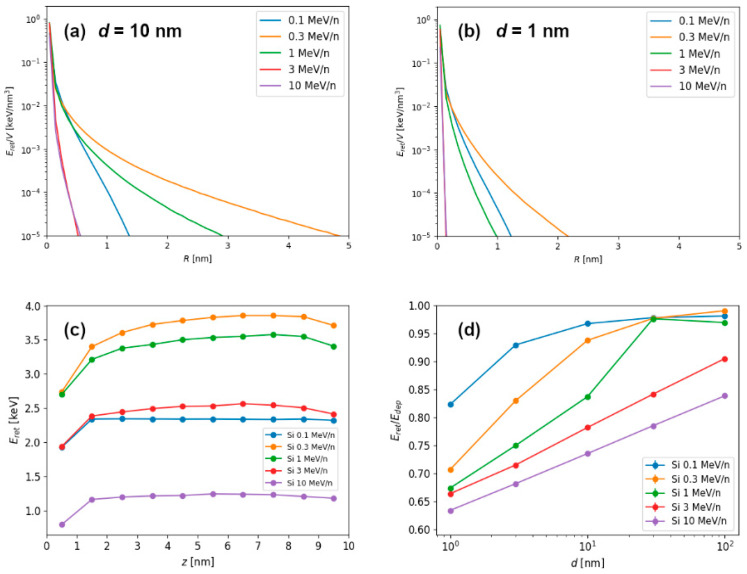
Simulation results for Si projectiles with kinetic energies between 0.1–10 MeV/n. Radial profiles of retained energy density for Si ions passing through (**a**) 10 nm thick and (**b**) 1 nm thin graphite target. (**c**) Depth profiles of retained energies within 10 nm thick target. (**d**) Energy retention for different target thicknesses and Si ion energies.

The final spatial retained energy density distribution, obtained from the layer of material from the middle of the 10 nm thick target (to avoid possible effects related to the surface proximity) is shown in Figure 3a for different kinetic energies of silicon ions. It shows well-known “velocity effect”, when slower velocity ions cause more localized and dense electronic excitations, while higher velocity ions cause propagation of excitation to larger volumes, resulting in smaller densities of deposited energy. To a lesser degree, the same is observed for the 1 nm thin target shown in Figure 3b. Again, radial density of retained energy is more localized around the ion impact point due to a higher percentage of deposited energy being lost via the electron emission. In addition to the radial profiles of retained energy density distribution, it is possible to integrate retained energy density in different layers of target material, and thus obtain depth profiles of retained energy, as shown in Figure 3c.

Results of investigation of different Si ion energies and target thicknesses are shown in Figure 3d. In general, the increase in the target thickness results in the increase of *energy retention* (i.e., ratio of retained and deposited energy). While 1 nm and 3 nm thin targets exhibit decreased energy retention, 30 nm thick target can already be considered as a bulk-like material. For the slowest ions, even the 10 nm target behaves very much like a bulk material. 

**Figure 4:**
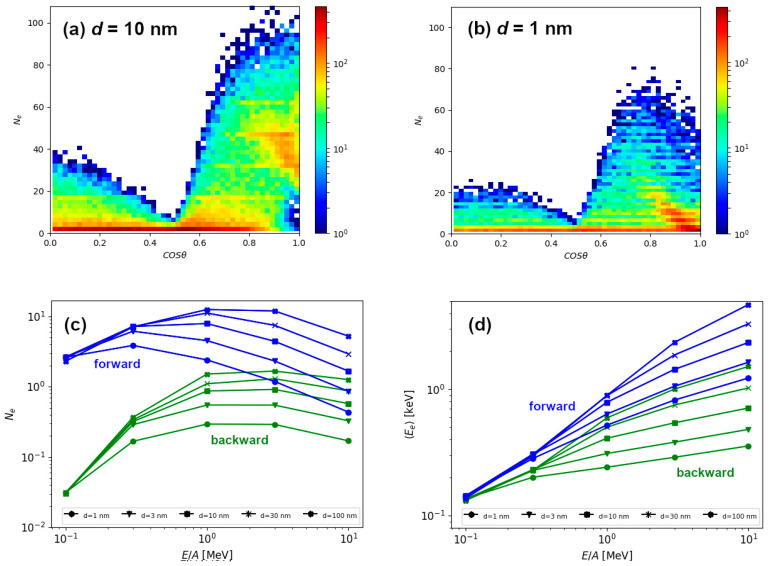
Distribution of emitted electrons from (**a**) 10 nm thick target and (**b**) 1 nm thin target, after 1 MeV/n silicon impact, as a function of the exit angle *θ*. The excessive content from the lowest *N_e_* bin has been ignored (**c**) Number of electrons and (**d**) average energy taken away via electron emission in forward and backward directions, for varying target thickness and Si ion energies.

To gain better understanding of the energy retention, we have investigated the number of emitted electrons and their average energies, in both forward and backward directions. Emission of electrons in backward direction is due to electron backscattering in the target. Forward moving electrons are those emitted from the exit surface of the target. Distribution of electrons depending on the *exit angle θ*, relative to the initial direction of 1 MeV/n Si ion is shown in Figure 4a for the 10 nm thick target and Figure 4b for the 1 nm thin target. In both cases, much more forward moving than backward moving electrons have been found. As shown in Figure 4c, a difference can be as large as an order of magnitude for slow Si ions, but this difference quickly decreases with the increasing ion energy. For the lowest energy, number of emitted electrons does not depend on the thickness of the target, but already at 1 MeV/n these differences are notable. Since the number of the emitted electrons decreases beyond Bragg peak, the number of emitted electrons is correlated with the ion electronic energy loss, i.e., with the density of the retained energy. Surprisingly, even for the thickest target, there exists a correlation in the number of emitted electrons with the target thickness for energies between 1–10 MeV/n, suggesting the electron excitations from deep within the material can still contribute to the process of emitting electrons. This explanation can be supported by average energy carried away by the emitted electron shown in Figure 4d. Instead of scaling with the ion energy loss, this graph shows strong correlation with the kinetic energy of the ion. This feature, along with quite high energy of emitted electrons (on average), indicate that many of the electrons emitted into the vacuum are primary electrons, i.e., the ones ejected by the energetic ion. For the non-relativistic ion of mass *M* and kinetic energy *T*, maximum kinematically allowed energy $E_{e}^{max}$ transferred to the electron of mass *m* (m≪M) is given by

(2) $$E_{e}^{max}=4\frac{m}{M}T$$

For example, in the case of 1 MeV/n Si ion, this maximum energy transfer is around 2 keV, quite close to the average value of the electron energy that lies between 0.5–1 keV in the case of 1 MeV/n Si ion irradiation (Figure 4d).

**Figure 5:**
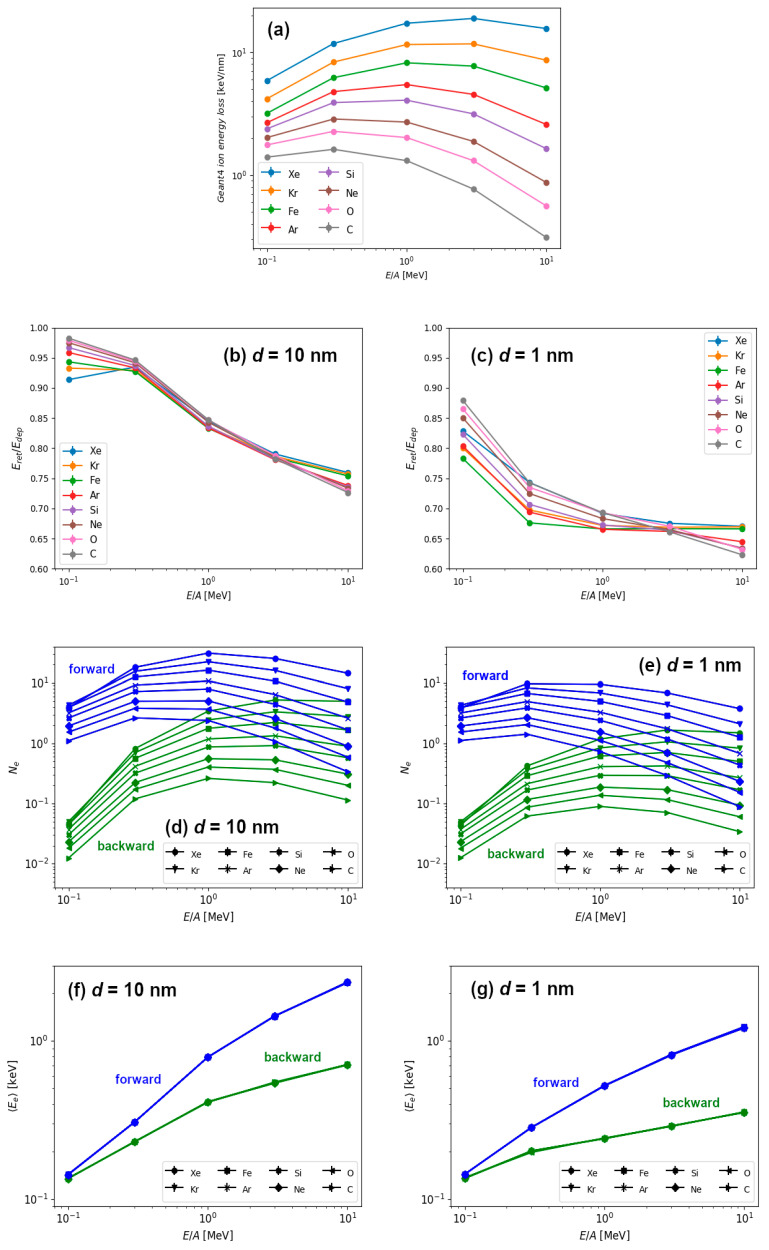
(**a**) Ion energy losses calculated using Geant4.10.05. Energy retention in the case of (**b**) 10 nm thick target and (**c**) 1 nm thin graphite target for different ions and different ion energies used in this work. Number of electrons emitted in forward and backward direction for the 10 nm thick (**d**) and 1 nm thin (**e**) graphite targets for different types of ions and energies. Average energy of emitted electrons for (**f**) 10 nm thick and (**g**) 1 nm thin targets for different types of ions and ion energies.

Finally, in Figure 5 we show the results for the energy retention and electron emission for different combinations of ion types and ion energies. These results are obtained for the 10 nm thick and 1 nm thin graphite targets. All ion types used in this study had kinetic energies between 0.1–10 MeV/n. This way, we were able to investigate irradiation parameters close to the Bragg peak (i.e., when the ion energy loss attains maximum value). For heavy ions such as iron, this occurs around ~1 MeV/n, and for lighter ions it shifts down to ~0.5 MeV/n. This trend in ion energy losses as calculated by Geant4.10.05 (Figure 5a) is in good agreement with the results from the SRIM code [6]. 

In Figure 5b,c we present the energy retention (ratio of retained and deposited energy) in graphite targets with two different thicknesses (10 nm and 1 nm) for all combinations of ion types and their kinetic energies. For the lowest energy ions (0.1 MeV/n and 0.3 MeV/n), almost all deposited energy remains within the thicker target, regardless of the ion type used. In these cases, when more than 90% energy is retained, target can be considered as a bulk one. As expected, for these slowest ions, there is a difference in the energy retention between 1 nm thin and 10 nm thick targets, when significantly less energy (between 80–90%) remains in thin target. Actually, it is true for any ion speed that the energy retention is lower in 1 nm thin than in 10 nm thick target. By increasing the ion energy, the energy retention decreases both for the 10 nm thick and 1 nm thin targets. As a result, for the highest energy of 10 MeV/n, up to 40% of deposited energy can be emitted by electrons in the case of 1 nm thin target, and up to 30% for the 10 nm thick target. 

Next, we have studied the number of emitted electrons as well as their average energies. The results are shown in Figure 5d,f for the 10 nm thick target and Figure 5e,g for the 1 nm thin target, respectively. The number of emitted electrons shows the same behaviour as the one previously displayed for silicon ions (Figure 4c). The number of forward emitted electrons scales well with electronic energy loss because it exhibits similar behaviour close to the Bragg peak. For a given ion speed, the number of emitted electrons increases with the mass of the ion which is roughly proportional to the ion energy loss. The number of backward emitted electrons shows a similar behaviour at high ion energies, but at low ion energies the number of emitted electrons falls drastically. We attribute this behaviour to the kinematics of the ion-electron and electron-electron collisions, when forward emitted electrons can be primary electrons, i.e., electrons ejected from the material directly by the ion. On the other hand, one or more collisions between electrons are needed for the production of backward emitted electrons, and this should be the reason for their decreased number. This would also imply that primary electrons carry away most of the energy deposited by the energetic ion. 

We find more evidence supporting this scenario in the results of average energy carried away by the electrons, as shown in Figure 5f,g. Here, reported emitted electron energies scale only with the specific kinetic energy (MeV/n), i.e., the ion speed, and not with the ion type. Therefore, only kinematics in ion-electron collision play a role, since every ion is much more massive than single electron. We conclude that primary electrons carry away most of the emitted energy, and average value of emitted electron energies even above 1 keV agrees well with this finding. The increased portion of the less energetic electrons emitted from 1 nm thin target can be easily understood, since only those ejected close to the surface can leave the target at larger exit angles. This is, of course, due to the fact that greater relative portion of the thin target can be considered as being close to the surface. This also correlates well with rapid decrease in the radial deposited energy profile for the thin target shown in Figure 3b.

## 4. Discussion

As shown in Figure 3d, energy release from the ion irradiated targets can be significant even for the 10 nm thick targets. This is found for ions having speed beyond the Bragg peak, which is located around 1 MeV/n for heavy ions. Since the electrons are mostly ejected forward, the influence of the energy release on surface modifications followed by the ion irradiation of very thick and bulk targets is not significant [12]. However, in thin targets irradiated by the energetic ions, we observe that energy release can be up to 40% of the total deposited energy, because the number of forward emitted electrons (carrying away deposited energy) can be an order of magnitude greater than the number of electrons emitted in backward direction. For the same reason, the effect of predominant energy release via the exit surface of the target can make nanomaterials such as nanoparticles [26] and nanowires [27] less susceptible to the energetic ion irradiation induced damage (via electronic energy loss) than surfaces of bulk materials. However, irradiation of the very thin targets by energetic ions can lead to significant modifications due to Coulomb explosion mechanism, when target structural instability is caused by the charge imbalance. This is still an unexplored topic, although in the case of graphene this was found not to be relevant due to the extreme electrical conductivity of this material, resulting in ultrafast charge neutralization [28].

**Figure 6:**
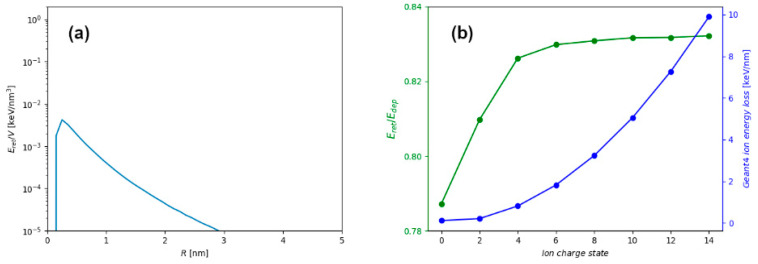
(**a**) Difference between radial distribution of retained energy densities obtained for irradiation of 10 nm thick and 1 nm thin targets with 1 MeV/n Si ion. (**b**) Ion energy loss and retention of energy for 1 MeV/n Si ion having different charge states.

Significant decrease of the retained energy in the case of thin targets is the most important, but not the only effect influencing early stages of the ion track formation in such targets. As shown in Figure 2a, radial profiles of retained energy for the 10 nm thick and 1 nm thin film are different, even for the same ion. This difference arises due to the proximity of the surface in thin target, which makes energetic electrons more difficult to contain within the target material. In thick target, the energetic electrons can traverse greater distances and carry energy further away from the ion trajectory, but still remain within the material. This finding is presented more clearly in Figure 6a, where the difference between radial distribution of retained energy densities for thick and thin targets is shown, with significant excess of electron excitation found in the whole volume of the thick target. Therefore, modelling of the later stages of ion track formation in thin targets (for example in thermal spike calculations [25]), should consider not only the missing energy, but also different radial energy distributions that are used as a model input.

Another important aspect of the energetic ion irradiation experiment is the use of the charge equilibrated ion beam when applied for surface and thin target modifications [29]. Since the ion electronic energy loss depends on the ion charge state, introduction of the stripper foil before the target ensures a charge equilibration, and consequently an ion impact which occurs with much higher ion energy loss. In Figure 6b, the ion energy loss and energy retention for 1 MeV/n Si ion and 10 nm thick graphite target are shown as a function of the ion charge state. In all simulation results presented so far, equilibrium charge state of the energetic ion has been assumed, and only in this case (1 MeV/n Si impact into 10 nm thick graphite), a charge-dependent stopping and the related energy retention have been explored. While the electronic energy-loss follows a known quadratic dependence on the ion charge state, the ratio of retained and deposited energy remains mostly unchanged. Only for the neutral projectile, when ion energy loss is very small but still not zero due to possible close encounters and direct collisions, this ratio drops significantly. However, this is not of much relevance for materials modifications because ion energy loss is almost negligible for such projectiles.

Finally, we consider the use of energetic ion irradiations for materials modifications when ion irradiation is done at non-normal incidence angles, in particular at grazing angles. This type of irradiation has been found to be very efficient in nanostructuring surfaces, thin films and 2D materials [20]. Grazing incidence irradiation by energetic ions produces long ion tracks on the material surface [30,31,32], and in the case of the 2D materials, such irradiation produces pores [15,33]. In both cases, stripping foil is not needed because energetic ions reach the equilibrium charge state within several nanometers. However, due to the proximity of the surface, such energetic ions travelling almost parallel to the surface can eject many electrons into the vacuum. This channel of energy dissipation could significantly affect the threshold for an ion track formation, similar to the case of the highly charged ion impacts into the surface [31,34]. The contribution of this and other ion track forming processes close to the surface remains to be investigated in the future.

## 5. Conclusions

Presented results show that the significant fraction of energy deposited into thin target by the impact of the energetic ion can be carried away by the emitted electrons. This is critically important in materials modification of the 2D materials such as graphene [21], but it can also have significant influence on energy deposition on surfaces [12] and within thin targets [18]. Actually, this feature can affect radiation hardness of not only thin targets, but also other nanomaterials such as nanoparticles and nanowires. For this reason, use of the stripper foils should be mandatory when the charge state of the ion delivered by the accelerator is significantly below its equilibrium value in the target material. This way, influence of the energy release can be counterbalanced by the increased electron energy loss due to higher charge state of the impinging ion.

In the present study we have examined an energy release from graphite target for a wide range of ion irradiation parameters (ion type, ion energy, and target thickness), and have shown that the energy release from the target depends primarily on the ion speed, and can be significant even for targets as thick as 10 nm. Most of the emitted energy is found to be released in the forward direction. As a consequence, high values of energy release yield low values of energy retention, especially for high energy ion irradiation of thin targets. The thinnest target examined in this work, having thickness of only 1 nm (corresponding to a three-layer graphene), has lowest energy retention of only 62% for 10 MeV/n carbon. We expect this value of energy retention to be even lower for a single-layer graphene, but more detailed atomistic simulations should be done to evaluate it precisely [21].

## Figures and Tables

**Table 1:** Ion irradiation parameters used in the present study, and ion energy losses obtained by Geant4.10.05 [1,2] and compared with SRIM [6] and CasP codes [7].

Ion Type	Ion Kinetic Energy (MeV)	Specific Energy (MeV/n)	SRIM dEe/dx (keV/nm)	SRIM dEn/dx (keV/nm)	CasP dEe/dx (keV/nm)	Geant4 dE/dx (keV/nm)
C	1.2	0.1	1.165	0.0084	1.148	1.4
	3.6	0.3	1.516	0.0034	1.403	1.62
	12	1	1.350	0.0012	1.189	1.31
	36	3	0.802	0.0005	0.759	0.77
	120	10	0.326	0.0002	0.322	0.31
O	1.6	0.1	1.499	0.0014	1.320	1.76
	4.8	0.3	2.127	0.0058	2.049	2.27
	16	1	1.999	0.0021	1.836	2.02
	48	3	1.294	0.0008	1.223	1.31
	160	10	0.574	0.0003	0.568	0.56
Ne	2	0.1	1.681	0.0216	1.487	2.02
	6	0.3	2.512	0.0089	2.609	2.86
	20	1	2.738	0.0032	2.599	2.70
	60	3	1.900	0.0012	1.793	1.88
	200	10	0.892	0.0004	0.876	0.87
Si	2.8	0.1	2.343	0.0401	2.270	2.38
	8.4	0.3	3.712	0.0166	3.935	3.89
	28	1	4.319	0.006	4.100	4.07
	84	3	3.106	0.0023	3.068	3.15
	280	10	1.585	0.0008	1.616	1.64
Ar	4	0.1	2.447	0.0635	2.542	2.67
	12	0.3	4.483	0.0265	5.285	4.78
	40	1	5.749	0.0097	5.643	5.45
	120	3	4.466	0.0038	4.443	4.54
	400	10	2.523	0.0013	2.502	2.58
Fe	5.6	0.1	3.088	0.1219	2.841	3.18
	16.8	0.3	6.141	0.0518	7.034	6.21
	56	1	8.514	0.0192	9.489	8.20
	168	3	7.622	0.0075	7.398	7.71
	560	10	4.630	0.0026	4.674	5.12
Kr	8.4	0.1	3.442	0.2158	3.410	4.18
	25.1	0.3	7.253	0.0935	8.992	8.32
	83.8	1	11.27	0.0351	13.051	11.55
	251.4	3	11.07	0.0138	12.034	11.70
	838	10	8.107	0.0048	7.788	8.61
Xe	13.1	0.1	4.390	0.4342	4.085	5.87
	39.4	0.3	9.502	0.1923	12.577	11.78
	131.3	1	16.54	0.0736	21.413	17.22
	393.9	3	18.73	0.0294	19.764	18.84
	1312.9	10	15.23	0.0104	14.6	15.58

## Data Availability

Data is available on request.
